# Ketogenic diet in the treatment of cancer – Where do we stand?

**DOI:** 10.1016/j.molmet.2019.06.026

**Published:** 2019-07-27

**Authors:** Daniela D. Weber, Sepideh Aminzadeh-Gohari, Julia Tulipan, Luca Catalano, René G. Feichtinger, Barbara Kofler

**Affiliations:** Research Program for Receptor Biochemistry and Tumor Metabolism, Department of Pediatrics, University Hospital of the Paracelsus Medical University, Müllner Hauptstraße 48, 5020, Salzburg, Austria

**Keywords:** Ketogenic diet, Tumorigenesis, Tumor metabolism, Adjuvant cancer therapy

## Abstract

**Background:**

Cancer is one of the greatest public health challenges worldwide, and we still lack complementary approaches to significantly enhance the efficacy of standard anticancer therapies. The ketogenic diet, a high-fat, low-carbohydrate diet with adequate amounts of protein, appears to sensitize most cancers to standard treatment by exploiting the reprogramed metabolism of cancer cells, making the diet a promising candidate as an adjuvant cancer therapy.

**Scope of review:**

To critically evaluate available preclinical and clinical evidence regarding the ketogenic diet in the context of cancer therapy. Furthermore, we highlight important mechanisms that could explain the potential antitumor effects of the ketogenic diet.

**Major conclusions:**

The ketogenic diet probably creates an unfavorable metabolic environment for cancer cells and thus can be regarded as a promising adjuvant as a patient-specific multifactorial therapy. The majority of preclinical and several clinical studies argue for the use of the ketogenic diet in combination with standard therapies based on its potential to enhance the antitumor effects of classic chemo- and radiotherapy, its overall good safety and tolerability and increase in quality of life. However, to further elucidate the mechanisms of the ketogenic diet as a therapy and evaluate its application in clinical practice, more molecular studies as well as uniformly controlled clinical trials are needed.

## Highlights

• Ketogenic diets (KDs) can enhance the efficacy of classical antitumor therapies.
• Effect of KDs on proliferation is tumor type dependent.
• Application of KDs to cancer patients is generally well tolerated.
• Low-carbohydrate and KDs increase quality of life of cancer patients.
• More standardized studies are needed before KDs can be advised for cancer patients.

## Graphical abstract

**Figure:**
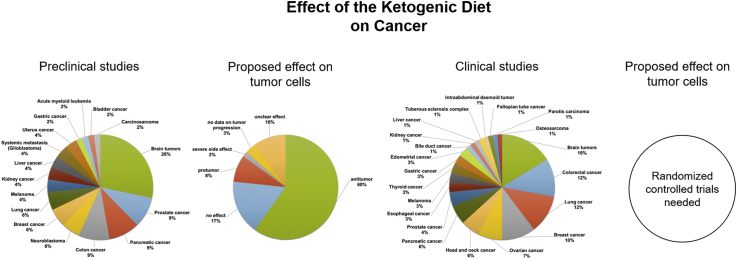

## Abbreviations

AA: amino acid
AcAc: acetoacetate
ACAT1: acetyl-CoA acetyltransferase
BDH1: d-β-hydroxybutyrate dehydrogenase
BHB: β-hydroxybutyrate
CA IX: carbonic anhydrase IX
HCA2: hydroxy-carboxylic acid receptor 2
HDAC: histone deacetylase
HDL: high density lipoprotein
HIF: hypoxia-inducible factor
HMG-CoA: β-hydroxy-β-methylglutaryl-CoA
IGF: insulin-like growth factor
IL: interleukin
KD: ketogenic diet
LDL: low density lipoprotein
MCT: medium-chain triglyceride
MAD: modified Atkins diet
NMDA: N-methyl-d-aspartate
OXCT1: 3-oxoacid CoA-transferase 1
OXPHOS: oxidative phosphorylation
PI3K: phosphatidylinositol-3 kinase
RCC: renal cell carcinoma
ROS: reactive oxygen species
SCOT: succinyl CoA: 3-oxoacid CoA transferase 1
TCA: tricarboxylic acid
TNF-α: tumor necrosis factor α
TSC: tuberous sclerosis complex
VEGF: vascular endothelial growth factor

## 1 Introduction

Worldwide, cancer is a major public health problem [1]. Although the war against cancer is being fought with the latest technologies on many fronts, there is still considerable room for improvement. In 2009, cancer-associated expenses in the EU amounted to 126 billion € [2].

In cancer cells, most energy comes from glucose even if oxygen is present. This shift from oxidative phosphorylation (OXPHOS) to glycolysis is called the Warburg effect [3]. Increased glycolysis and diminished tricarboxylic acid (TCA) cycle activity and OXPHOS are seen very early in tumorigenesis and constitute one of the hallmarks of cancer [4].

The ketogenic diet (KD) is a promising opportunity to target these metabolic alterations in tumor cells. Recent research shows that the KD potentially has a tumor growth-limiting effect, protects healthy cells from damage by chemotherapy or radiation, accelerates chemotherapeutic toxicity toward cancer cells [5], [6], and lowers inflammation [7]. Moreover, compared to anticancer drugs and standard treatments, the KD is inexpensive, fairly easy to implement (numerous good recipes are available in books and via the internet), and well tolerated [8], [9].

In the present review, we summarize the fundamentals of the KD, its proposed antitumor mechanisms, and currently available evidence from preclinical and clinical studies concerning efficacy. Finally, we discuss the future role of the KD as adjuvant therapy.

## 2 Fundamentals of the ketogenic diet

The KD is a high-fat, low-carbohydrate diet with adequate protein and calories originally developed in the 1920s as a treatment for intractable epilepsy [10]. At that time, ketone bodies were found in the blood of subjects on a starvation diet or a diet extremely low in carbohydrates [11]. Furthermore, it was proposed that the benefits of fasting could be obtained if the levels of ketone bodies could be elevated by other means [10]. Therefore, a new diet regime intended to mimic the effects of fasting was developed and termed the “ketogenic diet” [10], [12].

### 2.1 Types of ketogenic diets

The traditional KD is a 4:1 formulation of fat content to carbohydrate plus protein [10]. A classic 4:1 KD delivers 90% of its calories from fat, 8% from protein and only 2% from carbohydrate. KDs of the 1920s and 1930s were extremely bland and restrictive diets and, therefore, prone to noncompliance. In recent years, alternative KD protocols have emerged, making adherence to the diet much easier. Other characteristics besides macronutrient composition are increasingly recognized as important factors for long-term adherence to and efficacy of the KD [13]. These characteristics include fatty acid composition and nutrient density. Alternatives to the traditional KD are, for example, a medium-chain triglyceride (MCT)-based KD and the Atkins diet.

Compared to long-chain triglycerides, MCTs are more rapidly absorbed into the bloodstream and oxidized for energy because of their ability to passively diffuse through membranes [14]. Another characteristic of MCTs is their unique ability to promote ketone body synthesis in the liver [15]. Thus, adding MCTs to a KD would allow significantly more carbohydrate to be included [16], [17].

The Atkins diet, designed in the 1970s by Dr. Robert Atkins for weight loss, is characterized by carbohydrate restriction and its emphasis on fat. It is very similar to the classic KD but does not restrict protein or calories. The main difference between the Atkins diet and the modified Atkins diet (MAD) is that the MAD strongly encourages high-fat foods, carbohydrate intake is more restrictive and weight loss is not the primary goal [18]. In 2012, a review of the MAD for epilepsy was published, concluding that the MAD is effective for seizure control and should be the diet of first choice in that patient population [19].

As already mentioned, other characteristics besides macronutrient composition are increasingly recognized as important factors for long-term adherence to and efficacy of the KD. For far too long the sole focus of dietary advice was on macronutrient composition. The categorization of food into protein, carbohydrate, and fat is not sufficient to describe a well-formulated diet regarding its micronutrient density and hormonal or inflammatory effects on the human body. Unfortunately, in the literature, no clear definition of a KD is provided, and many studies define a KD as any diet which leads to an increase in blood ketones, for example diets in which no more than 50% of total calories are from fat [20]. In contrast, clinically used KDs mainly have a fat to carbohydrate and protein ratio of at least 2:1 to 3:1, meaning that the percentage of calories from fat is a minimum of 80%. The MAD can be considered as a mild KD. Thus, clear differentiation of low-carbohydrate diets and KDs cannot be made. In this paper, we review studies which use the terms KD and cancer. In the summary tables, we include the composition of the diets as far as provided by the studies (Table 1, Table 2).

**Table 1:** Preclinical studies reporting the effect of the KD on tumor progression and survival.

Tumor type	Animal model	Cell lines	KD ratio	Study groups	Glucose and ketone levels	Major outcome of the KD groups	Proposed effect on cancer cells	Ref.
Glioblastoma	athymic nude mice	T98G, U87MG, NIH-3T3, A172, LNT-229, U251MG	3:1	CD, KD	↔ glucose, ↑ BHB	KD: ↔ TP, ↔ survival	no effect	[105]
	athymic nude mice	U87MG	3:1	CD ± CT, KD ± CT	↔ glucose, ↑ BHB	KD: ↔ TP, ↔ survival KD + CT: ↔ TP, ↑ survival	no effect of KD alone; enhanced survival of KD + CT vs. CD + CT	[52]
	albino C57BL/6 mice	GL261-Luc2	4:1	CD, KD	↓ glucose, ↑ BHB	↓ hypoxic response, ↓ tumor microvasculature gene expression and peritumoral edema	no data on TP reported	[149]
	albino C57BL/6 mice	GL261-Luc2	4:1	CD, KD	↓ glucose, ↑ BHB	KD: ↓ TP, ↑ survival	antitumor	[153]
	albino C57BL/6 mice	GL261-Luc2	4:1	CD ± RT, KD ± RT	↓ glucose, ↑ BHB	KD ± RT: ↓ TP, ↑ survival	antitumor; additive effect of KD and RT	[54]
	Fischer rats	RG2, 9L	4:1	CR-CD, CR-KD	↓ glucose, ↑ BHB	CR-KD: ↔ TP, ↔ survival	no effect	[65]
	VM/Dk mice	VM-M3	4:1	CD, CR-KD, CR-KD + oxaloacetate and/or HBOT and/or CT	↓ glucose, ↑ BHB	CR-KD + oxaloacetate and/or HBOT and/or CT: ↑ survival	no effect of CR-KD compared to CR-CD; antitumor effect due to combination of therapies with CR-KD	[183]
	C57BL/6J; BALBc/J-SCID mice	U87-MG	4:1	CD, KD, CR-KD	KD: ↔ glucose, ↑ BHB CR-KD: ↓ glucose, ↑ BHB	KD: ↔ TP, ↔ survival CR-KD: ↓ TP, ↑ survival	effect not clear, because CR-CD group is missing	[106]
	VM/Dk mice	VM-M3	4:1	CD ± DON, CR-KD ± DON	CR-KD ± DON: ↓ glucose, ↑ BHB	CR-KD ± DON: ↓ TP, ↑ survival	effect not clear, because CR-CD group is missing; additive effect of CR-KD and DON	[184]
	NOD SCID mice	primary cell lines	0.7:1, 6:1	CD, HFLC, KD	↓ glucose, ↑ BHB	HFLC, KD: ↓ TP, ↑ survival	antitumor	[60]
	C57BL/6 mice	GL261	8:1	CD, KD	↔ glucose, ↑ BHB	KD: ↓ TP, ↑ survival	antitumor	[161], [162]
Astrocytoma	C57BL/6J; BALBc/J-SCID mice	CT-2A	4:1	CD, KD, CR-KD	KD: ↔ glucose, ↑ BHB CR-KD: ↓ glucose, ↑ BHB	KD: ↔ TP, ↔ survival CR-KD: ↓ TP, ↑ survival	effect not clear, because CR-CD group is missing	[106]
	C57BL/6J mice	CT-2A	4:1	CD ± DON, CR-KD ± DON	not specified	CR-KD ± DON: ↓ TP, ↑ survival	effect not clear, because CR-CD group is missing; additive effect of CR-KD and DON	[184]
	C57BL/6J mice	CT-2A	5:1	CD ± 2-DG, CR-KD ± 2-DG	not specified	CR-KD ± 2-DG: ↓ TP CR-KD + 2-DG: ↓ survival	effect not clear, because CR-CD group is missing; additive effect of KD and 2-DG on tumor weight	[185]
	C57BL/6J mice	CT-2A	5:1	CD, CR-CD, KD, CR-KD	KD: ↔ glucose, ↑ BHB CR-KD: ↓ glucose, ↑ BHB	KD, CR-KD: ↔ TP	no effect of KD; antitumor effect was based on CR	[98]
Medullo-blastoma	*Ptch1*^*+/-*^*Trp53*^*−/−*^ mice	spontaneous tumor development	4:1	CD, KD	not specified	KD: ↔ TP, ↔ survival	no effect (preventive)	[97]
	NOD SCID mice	Medulloblastoma from *Ptch1*^*+/-*^*Trp53*^*−/−*^ mice	6:1	CD, KD	↓ glucose, ↑ BHB	KD: ↔ TP	no effect	
Prostate cancer	SCID mice	LAPC-4	2:1	CD, KD	↑ glucose, ↑ BHB	KD: ↓ TP, ↑ survival	antitumor	[186]
	Fox Chase SCID mice	LNCaP	2:1	CD, KD	↔ glucose, ↑ BHB	KD: ↓ TP, ↑ survival	antitumor	[96]
	athymic nude mice	LAPC-4	2:1	CD ± MCT1 inhibitor, KD ± MCT1 inhibitor	↔ glucose	KD ± MCT1 inhibitor: ↔ TP and survival	trend to ↓ TP and ↑ survival in KD groups; KD significantly ↓ necrosis	[107]
	SCID mice	LAPC-4	0.8:1, 1.2:1, 2:1	CD ± castration, 20% CHO, 10% CHO, NCKD	↓ glucose, ↔ BHB	KDs: ↓ TP, ↔ survival	antitumor	[187]
	transgenic Hi-Myc mice	spontaneous tumor development	2:1	CD, KD	not specified	KD: ↑ TP	protumor (preventive)	[188]
Pancreatic cancer	athymic nude mice	S2-013	2.1	CD, KD	↓ glucose, ↑ BHB	KD: ↓ TP	antitumor	[47]
	nu/nu mice	PANC-1	3:1	CD, KD	↓ glucose, ↑ BHB	KD: ↓ TP, ↑ survival	antitumor	[62]
	athymic nude mice	MIA PaCa-2	4:1	CD ± RT, KD ± RT	↓ glucose, ↑ BHB	KD: ↔ TP, ↔ survival KD + RT: ↓ TP, ↑ survival	no effect of KD alone; enhanced antitumor effect of KD + RT vs. CD + RT	[55]
	C57BL/6 mice	Pan02, Pan02-LDH-knock down	6:1	CD, KD	↔ glucose	KD: ↔ TP	trend to ↓ tumor size in KD groups; ↑ antitumor immune response due to KD	[108]
	C57BL/6 mice	KPC K8484, K8082	6:1	CD ± PI3K inhibitors, KD ± PI3K inhibitors	↑ BHB	KD: ↔ TP, ↔ survival KD + PI3K inhibitors: ↓ TP, ↑ survival	no effect of KD alone; enhanced antitumor effect of KD + PI3K inhibitors vs. CD + PI3K inhibitors	[56]
Colon cancer	NMR1 mice	MAC16	1:1, 2:1	CD, 68% fat KD ± 12 mg BHB, 80% fat KD ± 12 mg BHB	↔ glucose, ↑ BHB	68% fat KD ± BHB: ↔ TP 80% fat KD ± BHB: ↓ TP	antitumor	[48]
	NMR1 mice	MAC16	2:1	CD, KD	↔ glucose, ↑ BHB	KD: ↓ TP	antitumor	[49]
	BALB/c nude mice	HCT-116	3:1	CD, LCT-KD, MCT-omega-3-KD	↔ glucose, ↑ BHB	LCT-KD and MCT-omega-3-KD: ↓ TP, ↑ survival	antitumor	[61]
	CDF1 mice	colon 26	3:1	CD, KD	↑ BHB	KD: ↓ TP	antitumor	[154]
	BALB/c mice	colon 26	4:1	CD, KD	↔ glucose, ↑ BHB	KD: ↔ TP, ↑ survival	antitumor	[189]
Neuroblastoma	CD-1 nude mice	SH-SY5Y (non-NMYC-amplified)	2:1	CD, CR-CD, KD, CR-KD	KD: ↔ glucose, ↑ BHB CR-KD: ↓ glucose, ↑ BHB	KD, CR-KD: ↓ TP, ↑ survival	antitumor; additive effect of KD and CR	[63]
		SK-N-BE(2) (NMYC-amplified)				KD: ↔ TP, ↔ survival CR-KD: ↓ TP, ↑ survival	no effect of KD alone, but enhanced effect of CR-KD vs. CR-CD	
	CD-1 nude mice	SH-SY5Y (non-NMYC-amplified)	2:1	CD, CD + CT, CR-CD + CT, KD + CT, CR-KD + CT	KD: ↔ glucose, ↔ BHB CR-KD: ↓ glucose, ↑ BHB	KD + CT, CR-KD + CT: ↓ TP, ↑ survival	antitumor effect of KD + CT; additive effect of KD + CT and CR	[50]
		SK-N-BE(2) (NMYC-amplified)			KD: ↔ glucose, ↑ BHB CR-KD: ↓ glucose, ↑ BHB	KD + CT: ↔ TP, ↔ survival CR-KD + CT: ↓ TP, ↑ survival	no effect of KD + CT vs. CD + CT, but enhanced antitumor effect due to CR	
	CD-1 nude mice	SH-SY5Y (non-NMYC-amplified)	8:1	CD + CT, LCT-KD + CT, MCT-KDs + CT	↓ glucose, ↑ BHB	LCT-KD + CT: ↓ TP, ↔ survival, MCT-KDs: ↓ TP, ↑ survival	antitumor	[51]
		SK-N-BE(2) (NMYC-amplified)						
Breast cancer	transgenic FVB MMTV-PyMT mice	spontaneous tumor development	4:1	CD, KD	not specified	KD: ↓ TP	antitumor (preventive)	[190]
	BALB/c mice	4T1	6:1	CD ± metformin, CR-KD ± metformin	↓ glucose	CR-KD ± metformin: ↓ TP	effect not clear, because CR-CD groups are missing; enhanced effect of CR-KD + metformin vs. CD + metformin	[176]
	C57BL/6 mice	ES-272	6:1	CD ± PI3K inhibitors, KD ± PI3K inhibitors	not specified	KD: ↔ TP; KD + PI3K inhibitors: ↓ TP	no effect of KD alone; enhanced antitumor effect of KD + PI3K inhibitors vs. CD + PI3K inhibitors	[56]
Lung cancer	C57BL/6 (*Fgf2*1 WT and KO) mice	LLC1	3:1, 8:1	low-fat diet (CD), regular protein KD, low protein KD	regular protein KD: ↔ glucose, ↑ BHB low protein KD: ↓ glucose, ↑ BHB	regular protein KD: ↔ TP low protein KD: ↓ TP	antitumor effect of low protein KD	[109]
	nu/nu mice	NCI-H292, A549	4:1	different experiments with different IR doses, but overall: CD ± RT, KD ± RT, CD + RT/CT, KD + RT/CT	↑ BHB	KD: ↔ TP, ↔ survival KD + RT: ↓ TP, ↑ survival; KD + RT/CT: ↓ TP, ↑ survival	no effect of KD alone; enhanced antitumor effect of KD and RT as well as KD, RT and CT	[53]
	cre-transgenic mice (C57BL/6J background)	Adeno-Cre virus: K-*Ras*^LSLG12Vgeo^; *p53*^lox/lox^	4:1	15–18 h fasting, 3 days KD	↓ glucose	↓ myocardial but not tumor FDG uptake	no data on TP reported	[191]
Melanoma	nu/nu mice	A375, A2058 (BRAF V600E)	4:1, 6:1	CD, KD	↓ glucose, ↔ BHB, ↑ AcAc	KD: ↑ TP	protumor	[67]
		SK-MEL-2 (NRAS Q61R), HMCB (NRAS Q61K)	6:1		↓ glucose, ↔ BHB, ↑ AcAc	KD: ↔ TP	no effect	
		PMWK (BRAF WT)	6:1		↔ BHB, ↑ AcAc	KD: ↔ TP	no effect	
	C57BL/6 mice	B16	pure oil	sucrose solution (CD) and vegetable oil (KD)	↓ glucose, ↑ BHB	KD: ↓ metastatic load	antitumor	[59]
Kidney cancer	CD-1 nude mice	786-O	8:1	CD, LCT-KD, MCT-KDs	LCT-KD: ↔ glucose, ↑ BHB MCT-KDs: ↔ glucose, ↔ BHB	LCT-KD and MCT-KDs: ↔ TP MCT-KD: ↓ survival	no significant effect of KDs, but trend to ↓ TP; severe body weight loss lead to ↓ survival in KD groups	[64]
	Eker (*Tsc2*^+/−^) rats	spontaneous tumor development	8:1	CD, KD	↓ glucose, ↑ BHB	KD: ↑ TP	protumor (preventive)	[192]
Liver cancer	C57BL/6N mice	DEN-induced hepatocellular carcinoma	4:1	CD, KD	↑ BHB	KD: ↔ TP	no effect	[193]
	C57BL/6N mice	DEN-induced hepatocellular carcinoma	5:1	low-fat/low-sucrose diet, KD, western diets, fructose diet	↔ glucose	KD and low-fat/low-sucrose diet: ↓ tumor burden vs. all high-sugar diets	antitumor (preventive)	[194]
Systemic metastasis	VM/Dk mice	VM-M3	1.5:1	CD, KD, KD + KE, KD + KE + HBOT	KD: ↔ glucose, ↔ BHB KD + KE, KD + KE + HBOT: ↓ glucose, ↑ BHB	KD, KD + KE, KD + KE + HBOT: ↓ TP, ↓ metastatic spread, ↑ survival	antitumor	[58]
	VM/Dk mice	VM-M3	4:1	CD ± HBOT, KD ± HBOT	↓ glucose, ↔ BHB	KD ± HBOT: ↓ TP, ↑ survival	antitumor; additive effect of KD and HBOT	[57]
Uterus cancer	nu/nu mice	HeLa	3:1	CD, KD	↓ glucose, ↑ BHB	KD: ↔ TP, ↓ survival	protumor	[62]
	nude mice	Patient derived xenograft	6:1	CD ± PI3K inhibitors, KD ± PI3K inhibitors	not specified	KD: ↔ TP KD + PI3K inhibitors: ↓ TP	no effect of KD alone; enhanced antitumor effect of KD + PI3K inhibitors vs. CD + PI3K inhibitors	[56]
Gastric cancer	NMRI nude mice	23132/87	3:1	CD, KD	↔ glucose, ↑ BHB	KD: ↓ TP, ↑ survival	antitumor	[46]
Acute myeloid leukemia	C57BL/6 mice	MLL-AF9 Ds-Red	6:1	CD ± PI3K inhibitors, KD ± PI3K inhibitors	not specified	KD: ↔ TP, ↓ survival KD + PI3K inhibitors: ↑ survival	protumor effect of KD alone; enhanced survival of KD + PI3K inhibitors vs. CD + PI3K inhibitors	[56]
Bladder cancer	nude mice	Patient derived xenograft	6:1	CD ± PI3K inhibitors, KD ± PI3K inhibitors	not specified	KD: ↓ TP; KD + PI3K inhibitors: ↓TP	antitumor; additive effect of KD and PI3K inhibitors	[56]
Walker carcino-sarcoma	Sprague–Dawley rat	Walker carcinosarcoma 256	2:1–3:1	CD ± 2-DG, KDs ± 2-DG	↓ glucose	KDs ± 2-DG: ↓ TP	antitumor; additive effect of KD and 2-DG	[195]

↑: increased, ↓: decreased, ↔ not altered, 2-DG: 2-deoxyglucose, AcAc: acetoacetate, BHB: β-hydroxybutyrate, CD: control diet, CHO: carbohydrate, CR-CD: calorie restricted control diet, CR-KD: calorie restricted ketogenic diet, CT: chemotherapy, DEN: diethylnitrosamine, DON: 6-diazo-5-oxo-l-norleucine, HBOT: hyperbaric oxygen therapy, IR: ionizing radiation, KD: ketogenic diet, KE: ketone ester, KO: knock out, LCT: long-chain triglyceride, LFD: low-fat diet, MCT: medium-chain triglyceride, MCT1: monocarboxylate transporter 1, NCKD: non carbohydrate ketogenic diet, PI3K: phosphatidylinositol-3 kinase, RT: radiotherapy, TP: tumor progression, WT: wild-type.

**Table 2:** Clinical studies in the context of the KD and cancer.

Cancer	Study group size (n)	Dietary intervention (n)	Study comple-tion (n)	Combined with tumor therapy (n)	Study duration	Metabolic changes	Major outcome	Effect on QoL	Ref.
Glioblastoma	1	CR-KD 20 g KetoCal® 4:1 + 10 g fat, 32 g protein, 10 g CHO, 600 kcal/day (1)	1/1	ST	14 days CR-KD; 5 months CR	↓ glucose ↑ ketosis ↓ body weight	after two months: complete response; ten weeks after suspension of CR: tumor recurred	not specified	[77]
Glioblastoma	20	KD 60 g CHO/day (20)	8/20	ST	6 + weeks	↔ glucose ↑ ketosis ↓ body weight	trend to longer PFS in individuals with stable ketosis (n = 8); 1 complete response, 5 PR	3 stopped KD because they felt that CHO restriction ↓ QoL	[52]
Glioblastoma	2	CR-KD 3:1, 20% CR/day (2)	1/2	no	3 months	↔ glucose ↑ ketosis ↓ body weight	TP in both patients	not specified	[76]
Glioblastoma	32	KD 50% kcal fat, 25% kcal CHO, 1.5 g/kg protein (17), CD (15)	9/17, 8/15	55 mg POH	3 months	↔ glucose ↑ ketosis ↔ body weight	KD group: 78% PR, 11% SD, 11% TP; CD group: 25% PR, 25% SD, 50% TP; ↓ tumor area in the KD group compared to baseline, not in CD group	not specified	[20]
Glioblastoma	1	CR-KD 4:1, 900 kcal/day (1)	1/1	CT + RT + several medications + HBOT	9 months	↓ glucose ↑ ketosis ↓ body weight	significant TR, patient continued a KD with 1500 kcal/day + therapy; after 20 months: further TR	not specified	[75]
Glioblastoma	53	KD 30–50 g CHO/day (5), CR-KD (1)	6/6	RT (4/6)	3–12 months	↓ glucose ↑ ketosis ↓ body weight	5 TP; patient on CR-KD showed no tumor recurrence 12 months post RT	not specified	[90]
Glioblastoma and gliomatosis cerebri	9	KD 4:1 (5), CD (4)	2/5, 4/4	ST (4/5, 4/4)	2–31 months	↑ ketosis	strict KD: 1 SD, 1 TP; detectable ketones in the brain intermittent KD: 3 TP CD: 2 SD, 2 TP	not specified	[131]
Glioma	172	modified KD 70% kcal fat, 20 g CHO/day (6)	4/6	ST	3 months	↑ ketosis ↔ body weight	modified KD was well tolerated; no data on TP	self-reported good QoL	[80]
Glioma	8	MAD 20 g CHO/day (8)	7/8	ST (3/8)	2–24 months	↓ body weight	↑ seizure control in brain tumor patients; at 13.2 months of follow-up all patients were alive	not specified	[73]
Advanced stage malignant astrocytoma	2 children	KD 70% kcal fat, 30% kcal CHO + protein (2)	2/2	ST	8 weeks	↓ glucose ↑ ketosis ↑ body weight	2 complete responses; ↓ glucose uptake at tumor site by an average of 21.8%; both patients remained in remission 5 and 4 years after diagnosis, respectively	substantial ↑ QoL of patient 1 + significant ↑ in mood and skill learning	[74]
Invasive rectal cancer	359	KD >= 40% kcal fat and <100 g/day glycemic load (48)	48/48	RT (18/48)	not specified	not specified	KD ↓ the risk of cancer specific death; minimal difference in the risk of cancer specific death between KD and KD + RT; KD + RT ↓ the risk of cancer specific death compared to other deaths	not specified	[196]
Breast cancer	1	strict KD + high dose vitamin D3, not further specified (1)	1/1	no	3 weeks	not specified	changes in biological markers of breast cancer (↓ HER2 and ↑ PgR expression)	not specified	[72]
Triple-negative breast cancer	1	KD, not further specified (1)	1/1	MSCT + HT + BHOT	6 months	↑ ketosis ↓ body weight	clinical, radiological and pathological complete response	self-reported ↑ QoL	[69]
Liver metastases from colorectal cancer	12	LTPN (6), GTPN (6)	6/6, 6/6	no	3 h	not specified	↔ FDG uptake in liver metastasis after LTPN compared to GTPN	not specified	[197]
Gastro-intestinal tract	27	LTPN (9), GTPN (9), oral CD (9)	9/9, 9/9, 9/9	no	14 days	↔ glucose ↔ body weight	number of replicating cells: GTPN 32.2% ↑, LTPN 24.3% ↓, CD 15% ↑	not specified	[86]
Intra-abdominal desmoid tumor	1	LTPN (1)	1/1	no	5 months	↓ glucose ↑ ketosis ↔ body weight	↔ tumor volume	not specified	[71]
Pancreato-biliary cancer	30	KD 1–2:1 (20), CD (10)	10/20, 9/10	no	10 + days	↑ ketosis ↓ fat mass, preserved lean mass	KD significantly ↑ energy intake, meal compliance and meal satisfaction rate after surgery; no data on TP	not specified	[87]
Lung and pancreatic cancer	9	KD 4:1 (9)	3/9	ST	5–6 weeks	↔ glucose ↑ ketosis	suboptimal compliance to KD; lung cancer: 1 TP + brain metastases, 1 unknown response pancreatic cancer: 1 biliary obstruction + sepsis	not specified	[55]
Non-small cell lung cancer	44	mild KD, avoidance of high CHO foods (44)	42/44	MSCT + HT + BHOT	6 months	not specified	at 6 months: 95.4% survival, 61.4% overall response rate, 15.9% SD, 22.7% TP after follow-up: mean OS of 42.9 months, PFS of 41.0 months	not specified	[83]
Tuberous sclerosis complex	5 (3 children)	KD 3–4:1 (5)	5/5	no	3 months-5.7 years	↑ ketosis	KD did not suppress tumor growth or induce tumor regression	not specified	[82]
Ovarian and endometrial cancer	73	KD 70% kcal fat, 30% kcal CHO + protein (37), CD (36)	25/37, 20/26	ST (7/25, 4/26)	3 months	↓ glucose ↑ ketosis ↓ fat mass, preserved lean mass	inverse association of BHB and IGF-1 levels; ↑ physical function, ↓ cravings for starch food and fast food fats; patients in the KD group without chemotherapy reported significantly ↑ energy at 12 weeks compared to baseline; no data on TP	KD does not diminish QoL, KD may even ↑ QoL	[84], [85]
Head and neck cancer	12	KD, not further specified (12)	12/12	not specified	4 days	not specified	↓ mean lactate concentration in the tumor tissue	not specified	[104]
Colorectal, breast, and head and neck cancer	85	fasting prior to RT + ketogenic breakfast (MCT drink + 10 g EAA) on RT days or full KD + 10 g EAA on RT days (22); CD (63)	20/22; 61/63	RT (9/20; 30/61) or RT + CT (11/20; 31/61)	35–40 days	↑ ketosis colorectal + breast cancer: ↓ fat mass, preserved lean mass head and neck cancer: ↑ body weight and lean mass	ongoing clinical phase I study: first results indicate significant favorable effects of the KD on cancer patients’ body composition	not specified	[88]
Malignant diseases*	5	KD via nasogastric tube, 70% kcal fat, 30% kcal CHO + protein suppl. with BHB salt (5)	5/5	not specified	7 days	↓ glucose ↑ ketosis ↑ body weight	cachectic patients ↑ body weight after 7 days; patients maintained in a positive N balance; no data on TP	not specified	[70]
Advanced metastatic tumors*	16	LCHF < 70 g CHO/day (16)	5/16	no	up to 3 months	↓ glucose ↑ ketosis ↓ body weight	5 SD, patients reported ↑ emotional functioning and ↓ insomnia	↔ QoL or ↓ QoL, which reflects advanced stage diseases	[79]
Advanced malignancies*	17	MAD 20–40 g CHO/day (11)	4/11	no	up to 4 months	↔ glucose ↑ ketosis ↓ body weight	after 4 weeks: 5 TP, 6 SD or PR, those 6 dieted further to week 8: 1 TP, 5 SD; 4 continued the diet until week 16 and showed SD or TR; ↑ survival in 3 melanoma and 1 lung cancer patient	slightly ↑ QoL	[78]
Any type*	12	KD 5% CHO/day (10)	10/10	no	26–28 days	↓ glucose ↑ ketosis ↓ body weight	5 SD, 1 PR, 4 TP; level of ketosis correlated with SD or PR; insulin levels correlated positively and negatively with glucose and BHB, respectively	not specified	[81]
Any type*	6	KD < 50 g CHO/day (6)	6/6	RT	32–73 days	↔ glucose ↑ ketosis ↓ fat mass, preserved lean mass	5 TR (early stage disease); 1 slight TP; KD administered during standard therapy is safe and might be helpful in preserving muscle mass	↔ QoL, patients felt good on the diet and all continued a low CHO diet or KD after RT	[8]
Any type*	78	full KD (7) and partial KD (6), not further specified	not specified	not specified	10 months	not specified	correlation between an improvement of the disease and fully adopting a KD; KD ↓ TKTL1 levels	not specified	[91]

↑: increased, ↓: decreased, ↔ not altered, *: for types of cancer please see original publication, BHB: β-hydroxybutyrate, CD: control diet, CHO: carbohydrate, CR: calorie restriction, CR-KD: calorie restricted ketogenic diet, CT: chemotherapy, EAA: essential amino acids, GTPN: glucose-based total parenteral nutrition, HBOT: hyperbaric oxygen therapy, HER2: human epidermal growth factor receptor 2, HT: hyperthermia, KD: ketogenic diet, LCHF: low-carbohydrate high-fat diet, LTPN: lipid-based total parenteral nutrition, MAD: modified Atkins diet, MSCT: metabolically supported chemotherapy, OS: overall survival, PFS: progression free survival, PgR: progesterone receptor, POH: perillyl alcohol, PR: partial response, QoL: quality of life, SD: stable disease, ST: standard therapy, TKTL1: transketolase-like-1, TP: tumor progression, TR: tumor regression.

### 2.2 Ketone body synthesis

Ketones are organic compounds that are mainly produced by mitochondria of hepatocytes, but also, to some extent, in the heart, gut, kidneys and brain [21], [22]. The three main ketone bodies are acetoacetate (AcAc), β-hydroxybutyrate (BHB) and acetone. As an energy substrate, only AcAc and BHB are important, of which the latter is the most abundant ketone body in the blood. Acetone is formed spontaneously and breathed off via the lungs or further metabolized to pyruvate, lactate, and acetate [23]. The predominant substrates for ketone synthesis are fatty acids, although a small proportion of ketones is synthesized from leucine and in phenylalanine-tyrosine metabolism [24]. Free fatty acids are transported from adipose tissue to the liver and undergo β-oxidation to form acetyl-CoA. Under high-glucose conditions, acetyl-CoA is further shuttled into the TCA cycle and then into the electron transport chain to release energy. Under low-glucose conditions, acetyl-CoA generated from increased β-oxidation accumulates and challenges the processing capacity of the TCA cycle. Under these circumstances, the activity of the TCA cycle is low because of the low number of intermediates. Consequently, two molecules of acetyl-CoA are used for ketone synthesis via acetoacyl-CoA and β-hydroxy-β-methylglutaryl-CoA (HMG-CoA) driven by the ketogenic enzymes thiolase and HMG-CoA synthase, respectively [22]. HMG-CoA lyase splits HMG-CoA to re-generate acetyl-CoA and form one molecule of AcAc, which is further reduced by BHB-dehydrogenase to BHB [22].

Key regulators of ketogenesis are the hormones insulin and glucagon. Insulin inhibits ketogenesis, whereas glucagon stimulates ketogenesis [22]. The regulatory metabolic pathway works via hormone-sensitive lipase and acetyl-CoA-carboxylase, as well as HMG-CoA synthase. Insulin reduces lipolysis via inhibition of hormone-sensitive lipase and lowers the amount of free fatty acids, the substrate of ketogenesis. Insulin stimulates acetyl-CoA-carboxylase which controls lipogenesis. Additionally, insulin inhibits mitochondrial HMG-CoA synthase, which is the rate-limiting step in ketogenesis [24].

### 2.3 Ketone body utilization

In extrahepatic tissues, ketone bodies are taken up by the monocarboxylate transporters. These are found throughout the whole body and transport not only ketones but also lactate and pyruvate across the plasma membrane [25]. Ketolysis is the process by which ketone bodies are converted back to acetyl-CoA. Acetyl-CoA is further oxidized via the TCA cycle and the electron transport chain. Ketolysis occurs in almost all extrahepatic tissues [22]. Ketolysis is facilitated by three mitochondrial enzymes, d-β-hydroxybutyrate dehydrogenase (BDH1), 3-oxoacid CoA-transferase 1 (OXCT1), and acetyl-CoA acetyltransferase (ACAT1) [26]. BDH1 catalyzes the interconversion of AcAc and BHB. OXCT1, also known as succinyl CoA: 3-oxoacid CoA transferase 1 (SCOT), catalyzes the transfer of coenzyme A from succinyl-CoA to AcAc, leading to the formation of acetoacetyl-CoA, and is the key enzyme of ketone body utilization. Acetoacetyl-CoA is further catalyzed by ACAT1 to form two molecules of acetyl-CoA, which can be shuttled into the TCA cycle and oxidized to produce ATP.

### 2.4 Energy production from ketones

Per each 2-carbon unit, BHB yields more energy than glucose. Ketone bodies are, therefore, considered metabolically more efficient than glucose [27]. Ketones play a vital role as energy substrates for peripheral organs and the brain, especially during times of starvation and in early childhood brain development [28], [29], [30]. After 3 days of fasting, 30–40% of total energy requirements are covered by ketones. Ketone bodies rapidly increase during the first 10 days of a fast and reach a plateau after about 30 days [31]. In particular, the heart, skeletal muscle, kidney, and brain start to use ketones early. During fasting or under a KD, when glucose levels are low, the brain receives 60–70% of its required energy from ketones. The liver lacks the rate-limiting enzyme SCOT to use ketones as energy [22]. Therefore, although the liver can produce ketones, it cannot use a significant amount of them. The shift from glucose to ketones and fatty acids as the main source of energy can take up to one week [32].

### 2.5 Effects of the ketogenic diet on metabolic parameters

Besides the traditional use of the KD in therapy-resistant epilepsy, patients with malfunctions in glucose metabolism such as glucose transporter 1, pyruvate carboxylase, or pyruvate dehydrogenase deficiency benefit from a KD [33], [34]. Evidence also supports the KD as a therapeutic option for obesity, type 2 diabetes, polycystic ovary syndrome, acne, neurological diseases such as Alzheimer's disease and Parkinson's, and cancer [33], [34]. Owing to its high fat content, there is a general concern among clinicians that a KD may cause deterioration of certain metabolic markers, especially cholesterol and triglycerides. However, studies point toward an overall improvement of body composition and blood parameters [35], [36], [37]. Biological markers associated with the metabolic syndrome such as high blood lipid and cholesterol levels, elevated blood glucose and insulin levels, and reduced insulin sensitivity have been shown to improve significantly in patients with atherogenic dyslipidemia who consumed a carbohydrate-restricted diet for 12 weeks [38]. A further study in type 2 diabetic patients who consumed a KD for 56 weeks reported a significant reduction in body weight, body mass index, total cholesterol, low density lipoprotein (LDL) cholesterol, triglycerides, and blood glucose levels, and a significant improvement of high density lipoprotein (HDL) cholesterol [39]. However, in epileptic children with refractory seizures who consumed a KD for six months, one study detected increased serum lipid and cholesterol levels [40], whereas other studies reported normal lipid profiles [41], [42], [43]. Importantly, none of the listed studies reported severe adverse effects of the KD. Based on the available evidence, there is no obvious reason why a low-carbohydrate diet or a balanced KD might be contraindicated in adults. However, especially in patients, use of the KD should be meticulously planned and monitored by physicians and qualified dieticians. Because the classic KD (ratio 4:1) and KDs based on artificial foods potentially lack vitamins and minerals such as vitamin B, vitamin D, calcium, and iron, adequate supplementation of these micronutrients is essential [44], [45].

## 3 Ketogenic diet in the treatment of cancer - where do we stand?

### 3.1 Methodology of the literature review

This is a literature review in which we highlight some major findings from preclinical and clinical studies which have used some variations of KD as a single or combination therapy in cancer. Table 1, Table 2 of the review contain all studies from 1979 to 2019 listed in PubMed for the search terms “ketogenic diet” and “cancer”. Primary outcome parameters were tumor size/weight or survival. Secondary outcome parameters were alterations in vascularization, glucose up-take at the site of the tumor, gene expression patterns, as well as changes of metabolic parameters. In terms of clinical studies, changes in body composition and tolerance of the KD also were considered as outcome parameters. In total, we found 87 studies including 30 clinical studies and 57 original studies for rodents. In Table 1, Table 2, we summarize the key findings of these studies related to tumor progression, effects on blood glucose levels, and ketosis. In addition, Table 2 includes effects on quality of life as reported in the human studies. In both Table 1, Table 2, we indicate any diet given to a control group as being a control diet (CD) regardless of whether it was standard rodent chow, a matched control diet in preclinical studies or a normal (high carbohydrate/fiber, low fat) diet in clinical studies.

### 3.2 Preclinical evidence

A growing number of preclinical studies suggests that dietary intervention with a KD is a potent anticancer therapy, albeit some studies reported protumor effects or severe side effects in certain cancer models (Table 1). In most preclinical studies, KD slowed tumor growth, prolonged the survival rate, delayed the initiation of tumors [46], and reversed the process of cancer-induced cachexia [47], [48], [49]. In multiple studies, the KD sensitized cancer cells to classic chemo- [50], [51], [52], [53] or radiotherapies [53], [54], [55]. Furthermore, a study on different mouse cancer models, including pancreatic, bladder, endometrial, and breast cancer as well as acute myeloid leukemia, indicated that the KD enhances the efficacy of targeted therapy, in particular phosphatidylinositol-3 kinase (PI3K) inhibitors, and overcomes drug resistance [56], suggesting that the KD could be part of a multimodal treatment regimen to improve the efficacy of classic cancer therapy.

Some preclinical studies investigated the effect of the KD on metastasis formation, indicating a metastasis reducing potential of the KD [57], [58], [59]. However, preclinical data on KD and metastasis is sparse and urgently needs further investigation.

Several studies addressed the importance of optimizing the composition of the KD to enhance its efficacy, by increasing the proportion of fat or supplementing with MCTs, omega-3 fatty acids or ketone esters [46], [51], [58], [60], [61].

Some studies suggest that a number of metabolic features such as OXPHOS deficiency and/or low levels of ketolytic enzyme expression in cancer cells might be able to predict the effectiveness of KDs in cancer therapy [26], [62]. However, tumors seem to respond differently to a KD despite sharing similar metabolic signatures. For instance, we observed that a KD successfully suppressed the growth of neuroblastoma with OXPHOS deficiency [50], [51], [63], whereas the same KD led to different results in renal cell carcinoma (RCC), even though it presents an energetic profile (OXPHOS deficiency) similar to that of neuroblastoma [64]. Furthermore, the KD had no effect on the growth rate of rat glioma irrespective of the ability of the tumor cells to transport and oxidize ketone bodies [65].

Based on preclinical observations, the efficacy of KDs could be influenced by cancer type or even subtype, genetic background, or a tumor-associated syndrome [66]. For example, we observed that the anti-neuroblastoma effect of a KD was considerably attenuated in SK-N-BE(2) neuroblastoma xenografts, which carry MYCN amplification, TP53 mutation (p.C135F) and chromosome 1p loss of heterozygosity, compared to SH-SY5Y xenografts which are TP53 wild-type, non-MYCN-amplified [51].

In addition, in a mouse model of melanoma, acceleration of proliferation in BRAF V600E-mutated melanoma cells upon treatment with a KD was observed, due to selectively increased activation of BRAF V600E mutant-dependent MEK1 signaling by the ketone body AcAc. In contrast, NRAS Q61K- and Q61R-mutated as well as BRAF wild-type melanoma cells were unaffected by the KD [67]. Mice bearing RCC exhibited dramatic weight loss and liver dysfunction in response to the KD, most likely due to having features of Stauffer's syndrome [64], indicating that in certain patients with RCC, KD could be contraindicated.

Therefore, it is very important to evaluate the effect of a KD in preclinical studies for every specific type of tumor before recommending it to cancer patients, taking into account the different genetic alterations and tumor-associated syndromes. Furthermore, it is important to pay attention to the mechanism behind the antitumor effects of the KD. Understanding the mechanisms of KD therapy could assist in predicting the success rates of KDs against different types of cancer.

In summary, 60% of the preclinical studies shown in Table 1 reported an antitumor effect of KDs, 17% did not detect an influence on tumor growth and 10% reported adverse or pro-proliferative effects. In 10% of the preclinical studies, a statement on the effect on cancer cells cannot be made due to the lack of proper control groups. 3% of the preclinical studies did not report data on tumor progression but investigated the effect of the KD on tumor microvasculature, gene expression or glucose up-take. Most of the studies were performed in glioblastoma models and no adverse effects were observed. Accordingly, a majority of clinical studies are currently being performed on patients with glioblastoma [https://clinicaltrials.gov/].

### 3.3 Clinical evidence

For many types of cancers, the combination of surgery, radiation, and chemotherapy is the gold standard of care [68]. However, in highly aggressive cancer types with poor prognosis, for example triple negative breast cancer, no effective standard therapy is available [69]. Hence, new approaches that enhance therapeutic efficacy are urgently needed. As indicated by preclinical evidence, the KD represents a novel therapeutic approach for certain types of cancers (Table 1). Our aim was to summarize and critically evaluate human studies examining KDs in the context of cancer (Table 2). Most of the presented data are from case reports [8], [69], [70], [71], [72], [73], [74], [75], [76], [77] or pilot/feasibility studies [52], [55], [78], [79], [80], [81], [82], [83] mostly focusing on safety and tolerability of the KD. Only one randomized controlled trial is available to date [84], [85]. Nevertheless, consistent findings include a moderate reduction of blood glucose levels, induction of ketosis, feasibility and tolerability of the KD as well as improvement in quality of life (Table 2). Moreover, and very importantly, none of the studies reported any serious adverse events or toxicity related to the KD (Table 2), supporting the safety of a KD intervention.

Despite the lack of randomized controlled trials with large patient cohorts, several individual observations that support the antitumor effects of KDs have been reported in humans (Table 2). For example, an excellent therapeutic response to a KD was noted in two pediatric patients with advanced-stage malignant astrocytoma. The diet was administered either after or in combination with standard therapy [74]. After eight weeks of KD, positron-emission tomography revealed an average decrease of 21.8% in glucose up-take at the tumor site in both children. One of the children exhibited significant improvement in mood and skill learning and continued the KD for twelve months, remaining free of disease progression. Both patients remained in remission for five and four years after diagnosis, respectively, with good quality of life. In a second example, a women with triple-negative breast cancer who received a combination of KD with metabolically supported chemotherapy, hyperthermia, and hyperbaric oxygen showed a complete clinical, radiological, and pathological response [69]. Moreover, a prospective feasibility trial applying the MAD to patients with advanced malignancies who were not receiving chemotherapy reported that four patients were stable or improved after sixteen weeks of dietary intervention [78]. Interestingly, among these four patients, three were diagnosed with melanoma. All three continued the MAD beyond sixteen weeks and significantly exceeded their expected lifespan, which is usually only three months. Furthermore, a study in recurrent glioblastoma patients investigated the effect of a KD in combination with intranasal perillyl alcohol [20]. After three months of combined therapy, the partial response rate was 77.8% and 25% in the KD and the control group, respectively. Moreover, 50% of patients in the control group showed progression of the tumor compared to 11.1% in the KD group. In contrast, other studies could not support the potential tumor suppressive effect of the KD (Table 2). For example, a retrospective analysis of tumor images of five patients with tuberous sclerosis complex (TSC) receiving a 3–4:1 KD for seizure control revealed no significant effect of the KD on the growth suppression of TSC-related tumors [82].

Besides direct effects on tumor growth, the KD has the potential to improve the overall health status of patients as well as quality of life. Thus, some studies reported an overall normalization or improvement of lipid profiles, including reduction of total cholesterol, LDL, and HDL cholesterol, in cancer patients on a KD [20], [79], [86]. Furthermore, the KD significantly reduced insulin levels, and an inverse association between BHB and insulin-like growth factor 1 (IGF-1) concentrations was described [85]. In two insulin-dependent diabetic cancer patients, insulin requirements decreased by 75% [79] and 100% [78], respectively. A liver biopsy of a patient with an intra-abdominal desmoid tumor receiving lipid-based total parenteral nutrition for five months did not reveal lipid accumulation within the liver. Regarding quality of life, stable quality of life to significant improvements have been described for several cancer patients consuming a KD [8], [69], [74], [78], [79], [80], [84]. Moreover, a study investigating the effects of a KD on physical function, perceived energy and food cravings in ovarian and endometrial cancer patients reported overall improved physical health and increased energy in women on a KD without chemotherapy [84]. The authors stated that the latter effect could be related to the less advanced disease stage of the patients not undergoing chemotherapy.

Overall, most studies reported a reduction of body weight in patients who adhered to the diet (Table 2). In this regard it has been shown that the KD reduced total fat mass but was sufficient to preserve lean mass [8], [85], [87], [88]. In cachectic patients, however, the KD induced weight gain, and patients maintained a positive nitrogen balance [70]. In malnourished pediatric patients, the KD was designed specifically to induce weight gain, and the children showed weight stabilization and improved nutrient and caloric intake [74].

The reason why some studies could not draw any conclusion regarding the efficacy of the KD in cancer patients was either due to a lack of power of the study or due to low adherence of cancer patients to the diet regime (Table 2). In most cases, however, low adherence was attributed to either poor tolerability of the KD associated with nausea, fatigue, or constipation or because patients stopped the diet because of tumor progression. Poor tolerability has been reported, for example, in a study with lung and pancreatic cancer patients because of suboptimal compliance to a 4:1 KD [55]. One patient experienced asymptomatic grade 4 hyperuricemia, and another patient experienced grade 3 dehydration. That study was seeking a therapeutic response and, therefore, was very strict in achieving compliance to a high ratio KD. Based on our experience and as shown in several studies (Table 2), stable ketosis can also be achieved in most patients with a KD 2:1, indicating that a 4:1 KD as used for therapy-resistant epilepsy might not be necessary in cancer patients. Klement et al. suggested three measures that could help to maximize compliance and ketosis: 1) frequent support by an experienced dietitian, 2) provision of KD formulas and meals, and 3) offering cooking classes [8]. Furthermore, to support compliance to the KD, strong commitment and cooperation from both the patient and his/her family are necessary to maintain dietary-induced ketosis. Side effects, such as micronutrient deficiencies, appetite loss, nausea, headaches, light-headedness, constipation, fatigue, hyperlipidemia, reduced vision, and weight loss, could be avoided or reduced when the KD is initiated slowly and adequately supplemented with vitamins and minerals [8], [44]. Potential adverse effects of the long-term use of KDs, as for example gastrointestinal pain or renal stones [89], are generally mild, associated more with MCT oils, and can be reduced if the KD is consumed in restricted amounts/restricted time frames of radiochemotherapy [8], [77]. Nevertheless, several studies report good tolerability of the KD in patients who adhere to the diet [8], [20], [52], [69], [70], [71], [73], [74], [77], [78], [79], [80], [83], [90], and the authors mostly concluded that the use of a KD in cancer patients is feasible and safe [8], [52], [73], [76], [78], [79], [80], [81], [84], [87], [88], [90], [91].

## 4 Potential mechanisms of the ketogenic diet in tumorigenesis

Antitumor effects of the ketone bodies AcAc and BHB have been demonstrated in several cancer cell lines *in vitro* [59], [92]. However, whether the antitumor properties of KDs are exclusively attributable to the antiproliferative effects of ketone bodies and low blood glucose levels seems rather unlikely. In the following sections, we describe potential mechanisms of action of KDs in tumorigenesis.

### 4.1 Ketogenic diet targets glucose metabolism of cancer cells

Most solid cancers share metabolic features such as increased glucose uptake and reliance on glycolysis. In the Warburg effect, cancer cells predominantly use glycolysis for energy production accompanied by the production of lactate, paradoxically even if sufficient oxygen for respiration is present [93]. Therefore, it could be hypothesized that the Warburg effect in cancer cells could be at least partially targeted by creating chronic metabolic stress due to low glucose supply provoked by dietary intervention with a KD and/or calorie restriction.

Numerous preclinical studies on different types of cancer demonstrate that the KD, particularly in combination with calorie restriction, reduces circulating blood glucose levels (Table 1). The reduction in glucose levels is accompanied by a reduction of insulin and/or IGF levels in the blood [56], [94], [95], [96], [97], [98]. The activation of insulin and/or IGF receptor signaling pathways contributes to tumorigenesis [99]. In this context, a study including 9778 patients identified hyperinsulinemia as a risk factor in cancer prognosis [100]. Clinical studies have demonstrated a reverse correlation between the level of ketosis and the levels of glucose, insulin and/or IGF-1 [81], [85], [101].

The insulin-activated enzyme PI3K frequently exhibits enhanced activity in different types of cancer, due to PI3K gene mutations. Thus, PI3K inhibitors are considered as potent anticancer drugs. However, clinical trials have shown that targeted PI3K drugs often cause hyperglycemia [102], leading to increased insulin levels and reactivation of the PI3K pathway, which ultimately results in treatment resistance. Recently, it was shown that the KD improved the efficacy of anti-PI3K treatment and drug resistance by limiting the acute glucose-insulin feedback induced by PI3K inhibitors, thus blocking this loop [56].

In most cancer cells, accumulation of lactic acid, the major product of aerobic glycolysis, is detected [103]. It was shown that after three days of a KD, the level of lactic acid was diminished in the tumor tissue of patients with head and neck cancer [104]. In addition, reduction of another prognostic aerobic glycolysis-related marker, transketolase-like-1, was reported in patients who strictly used the KD [91].

Taken together, reduction of blood glucose seems to be a contributing factor in the effectiveness of the KD against cancer growth. Some preclinical studies showed that *ad libitum* KD, which failed to reduce the blood glucose level, was not able to reduce tumor growth [50], [52], [63], [98], [105], [106], [107], [108], [109], while additional calorie restriction or increasing the KD ratio led to both significant blood glucose reduction and tumor growth suppression [50], [63], [98], [106], [109]. Interestingly, in two preclinical studies, medulloblastoma and glioma did not respond to KD therapy, even though the KD (4:1) significantly reduced blood glucose [65], [97] and insulin levels [97].

### 4.2 Ketogenic diet targets mitochondrial metabolism of cancer cells

Some tumor entities are not able to properly respire due to a dysfunctional OXPHOS system. The mode of downregulation of OXPHOS can differ in different types of cancer. Thus, some tumors show a reduction of mitochondrial mass, others a reduction of all OXPHOS complexes and some, such as paragangliomas and oncocytic tumors, have pathogenic mutations in OXPHOS genes [110], [111], [112], [113], [114]. Tumors with dysfunctional mitochondria or decreased mitochondrial activity seem to compensate their energy requirements by aerobic fermentation [115]. Replacing glucose by ketone bodies requires that the tumors have functional mitochondria to be able to use ketone bodies efficiently for growth and survival. Thus, tumors with dysfunctional or low levels of mitochondria might suffer from high metabolic energy stress triggered by a KD [51], [60], [115], [116]. Analysis of the cellular energy sensor AMP-activated protein kinase (AMPK) in neuroblastoma tumors revealed that the KD increased the levels of activated AMPK [51].

On the other hand, tumor mitochondria can possess high activity in terms of respiration and energy production [117], [118], [119]. The question is whether the KD may also target tumors that have functional mitochondria. Rapidly growing tumors develop hypoxic areas in which oxygen supply is sparse [117]. Due to the capability of tumor cells to metabolize ketone bodies solely if enough oxygen is available [120], tumor cells at hypoxic sites would fail to produce energy from ketone bodies even though these cells have functional mitochondria.

The three mitochondrial enzymes SCOT, BDH1, and ACAT1 are key players in ketone body utilization. Thus, therapeutic efficacy might be influenced by the expression of these enzymes. For example, neuroblastoma and pancreatic cell lines and mouse xenografts with very low or no SCOT expression can be targeted by ketone bodies and KD [62], [63], [121]. In a recent clinical trial, differential expression of ketolytic enzymes (including BDH1 and OXCT1) was described in gliomas. The authors hypothesized that patients with low or very low expression of BDH1 and OXCT1 in malignant gliomas may respond better to KD therapy than patients with gliomas that express higher levels of ketolytic enzymes [26], [62]. In contrast, it has been shown that cancer cells of different origin can indeed take up and metabolize ketone bodies [122], [123], [124], [125]. *In vitro* analyses of several different breast cancer cell lines revealed that physiologic concentrations of ketone bodies did not reduce cell proliferation independent of the expression level of ketolytic enzymes [126]. Moreover, in a rat model of glioma, where the tumor cells were competent in the transport and oxidation of ketone bodies, a KD had no effect on cancer growth [65]. Taken together, it is still unclear whether ketone bodies play a major causal role in the antitumor effect of KDs.

### 4.3 Ketogenic diet targets amino acid metabolism of cancer cells

Based on the results of several animal model studies, the KD alters amino acid (AA) metabolism and urea cycle metabolites [51], [65], [127], [128], [129], [130]. The most consistent and pronounced changes observed were decreased blood levels of most essential AAs in mouse or rat [51], [128], [130]. In addition, in different studies, alterations of metabolism of other AAs such as glutamate/glutamine, glycine, serine, proline, tryptophan, and aspartate were reported [51], [65], [127], [128], [129], [130]. Douris et al. concluded that the KD led to down-regulation of AA catabolic processes in mice to conserve AA levels [128].

In a preclinical neuroblastoma model, reductions of essential AAs and urea cycle metabolites in plasma and tumors were induced by low-protein KDs, whereas the plasma levels of serine, glycine and glutamine were elevated [51]. Mouse models of glioma administered a KD also showed higher levels of glutamate in the cortex and tumor tissue [65]. In agreement, a clinical study reported increased levels of glutamine and/or glutamate in some patients with brain tumors after administration of a KD [131]. Considering the dependence of a range of tumor cells on glutamine and glutamate metabolism, it is surprising that the observed elevated levels of these AAs did not trigger tumor proliferation.

The impact of the KD on down-regulation of essential AAs most likely contributes to the inhibition of tumor growth, but this needs further investigation. It can be postulated that the reduction of essential AAs might result from relatively low amounts of protein in the KD. In contrast, Aminzadeh-Gohari et al. found neither a reduction of plasma essential AAs nor a reduction in tumor growth in mice fed a control diet containing the same low amount of protein as the KD [51].

### 4.4 Ketone bodies as signaling molecules

Extensive information concerning the molecular targets of ketone bodies comes from studies in brain malignancies and brain injury models. The obtained results are in general discussed with regard to the anti-seizure activity of ketones. However, some of the potential targets and mechanisms might explain the mode of action of the KD in tumor therapy. In addition, the downstream products of BHB metabolism, including acetyl-CoA, succinyl-CoA, and nicotinamide adenine dinucleotide, have signaling activities [132].

Modulation of N-methyl-d-aspartate (NMDA) signaling by BHB has been shown in several studies [133], [134]. The ketone bodies acetone and BHB inhibit the function of specific NMDA receptors in *Xenopus* oocytes. Acetone enhances, while BHB inhibits, α1 glycine and α1β2γ2S GABA_A_ receptor function at physiologically relevant concentrations [135]. NMDA receptor expression has been observed in various types of cancer, along with other glutamate receptors, but functional validation has largely been limited to demonstrating the effect of receptor blockade on cell survival [136].

Hydroxy-carboxylic acid receptor 2 (HCA2) is a G protein-coupled receptor that is activated by BHB. HCA2 can activate specific macrophages, which have neuroprotective effects [137]. Activation of retinal HCA2 by systemic BHB inhibits diabetic retinal damage through reduction of endoplasmic reticulum stress and the NLRP3 inflammasome [138]. Interestingly, HCA2 was described as a tumor suppressor. Decreased synthesis of BHB suppresses signaling via the HCA2 receptor. Therefore, low levels of BHB attenuate the tumor-suppressing function of HCA2 in colon [139]. Recent studies have extended the tumor-suppressive function of the receptor beyond the colon, as HCA2 suppresses mammary tumorigenesis in a mouse model of breast cancer [140].

Mitochondrial membrane potential depends on several factors, an important one being the balanced presence of anti-apoptotic Bcl-2 and Bcl-xl and pro-apoptotic Bad and Bax proteins. Phosphorylation of Bad at Ser112 and Ser136 promotes its binding to 14-3-3 proteins, sequestering Bad away from the mitochondrial membrane [141]. A KD increased the phosphorylation of Bad Ser136 and the interaction between Bad and 14-3-3, actions which may underlie the diet's neuroprotective properties against kainic acid-induced status epilepticus [142]. In the early 1970s, Kerr et al. linked apoptosis to the elimination of malignant cells, hyperplasia and tumor progression [143], [144]. Furthermore, reduced apoptosis or its resistance plays a fundamental role in carcinogenesis.

In contrast to the antitumor effects of ketone bodies described so far, it has been reported that ketone bodies can behave as onco-metabolites and that enzymes involved in ketogenesis or ketolysis are metabolic oncogenes. This was shown *in vivo* and *in vitro* using breast cancer xenografts and co-cultures of breast cancer cell lines and immortalized fibroblasts [145]. Moreover, the ketogenic enzyme HMG-CoA lyase is upregulated in BRAF V600E-expressing human primary melanoma and hairy cell leukemia cells. Active BRAF upregulates the HMG-CoA product AcAc, which selectively enhances binding of BRAF V600E but not BRAF wild-type to MEK1 to promote activation of MEK-ERK signaling to stimulate tumor growth [67], [146].

### 4.5 Ketogenic diet targets angiogenesis, vascularization and the tumor environment

Various interactions of cancer cells with their environment contribute to tumor progression and metastasis. Tumor cells are capable of creating a suitable microenvironment for their development and invasion by enhancing vascularization, repressing the immune response, and inducting inflammation.

A high level of angiogenesis is a key risk factor for progression in cancer. Therefore, anti-neovascularization strategies are considered one option for combating cancer [147]. In mouse models of brain cancer, neuroblastoma, gastric cancer, and liver cancer, a calorie-restricted or *ad libitum* KD either alone or in combination with chemotherapy reduced the level of vascularization and resulted in a reduction of intra-tumoral hemorrhage [46], [51], [58], [106], [148], [149]. Even though these studies suggest that KDs are anti-angiogenic, the mechanism behind them has not been fully characterized. During tumor progression, many angiogenic activators support the process of angiogenesis, such as vascular endothelial growth factors (VEGFs), interleukin 8 (IL-8), tumor necrosis factor α (TNF-α) and hypoxia-inducible factors (HIFs) [147]. In mouse glioma models, a KD or caloric restriction induced the reduction of the tumor microvasculature, accompanied by significant reduction of HIF-1α and VEGF receptor 2 levels [149].

Rapidly proliferating cancer cells often experience an imbalance between high oxygen demand and inadequate oxygen supply, resulting in a hypoxic environment. HIFs and carbonic anhydrase IX (CA IX) are commonly used markers of hypoxia that regulate cellular hypoxic responses [150], [151]. In a glioma mouse model, an *ad libitum* KD led to significantly reduced HIF-1α and CA IX levels in the tumor [149].

Inflammation and innate immunity are markedly associated with tumorigenesis [152]. Studies in mouse models of glioma and pancreatic cancer have shown that KDs improve the immune response against cancer progression [108], [153]. Moreover, a variety of *in vitro* and *in vivo* studies provides evidence that the KD and ketone bodies (especially BHB) have an anti-inflammatory effect via suppression of the NLRP3 inflammasome and reduction of inflammatory factors like TNF-α, IL-1, -6 and -18 and prostaglandin E2 [7], [98], [154], [155], [156], [157], [158], [159], [160]. In a colon cancer mouse model, a KD suppressed the elevation of plasma IL-6 and subsequent progression of inflammation [154]. NLRP3 is a multi-protein complex that controls the activation of caspase-1 and the release of the pro-inflammatory cytokines IL-1β and IL-18 in macrophages. Youm and colleagues found that BHB, but not AcAc, inhibits NLRP3 inflammasome assembly [7]. In an *in vitro* study, BHB suppressed the migration of glioma cells by inhibition of the NLRP3 inflammasome [158]. Further evidence comes from numerous other diseases and disease models highlighting the effect of BHB on NLRP3 [156], [157].

### 4.6 Ketogenic diet regulates gene expression

A variety of studies showed that KDs modulate gene expression. Gene expression profiling studies in glioma indicate that KDs can reverse the patterns of gene expression in tumors to those of non-tumor cells [161], [162]. The normalization of gene expression by the KD could be a result of the elevated levels of ketone bodies produced under the diet. In the kidneys of aged rats, BHB reduced aging-related inflammation through upregulation of forkhead transcription factor 1 and its target genes [160].

In addition, BHB has been shown to inhibit histone deacetylases (HDACs) [163], [164], [165]. HDACs are enzymes that remove acetyl groups from lysine residues on histones and other proteins such as transcription factors and enzymes. Deacetylation of histones loosens the tight wrapping of DNA, thus enabling gene transcription, whereas deacetylation of transcription factors or enzymes can increase or decrease their activity. Shimazu et al. found that BHB inhibits HDAC1, HDAC3, and HDAC4, supporting the physiological relevance of this mechanism of action [163]. HDACs are involved in multiple different stages of cancer. Aberrant expression of classic HDACs has been linked to a variety of malignancies, including solid and hematological tumors. In most cases, a high level of HDACs is associated with advanced disease and poor outcomes in patients [166]. It was shown that a KD increases overall lysine acetylation levels as well as acetylation of p53, the most frequently affected tumor suppressor in cancer. The authors hypothesized that p53 hyperacetylation and stabilization may also have contributed to the marked decrease in cancer incidence in the mice fed a KD for a long period [167]. Interestingly, posttranslational modification of histones via lysine β-hydroxybutyrylation has been reported recently [168]. Histone β-hydroxybutyrylation thus represents a new epigenetic regulatory mark that couples metabolism to gene expression. However, Chriett et al. were not able to confirm the inhibitory effect of BHB on HDACs, either *in vivo* or *in vitro.* They reported that butyrate, a short chain fatty acid structurally similar to BHB, is a strong inhibitor of HDACs, which resulted in an anti-inflammatory effect [164].

The KD also affects DNA methylation [169]. However, it is likely that ketone bodies or KDs influence the global levels of many more posttranslational modifications than reported so far. It was estimated that over 200 different posttranslational modifications exist, and identification of the specific targets might open a new frontier in fighting a variety of diseases, including cancer.

### 4.7 Ketogenic diet and ROS production

Reprograming of metabolism in cancer cells, mitochondrial dysfunction, and microenvironment-associated instability all induce continuous and elevated production of reactive oxygen species (ROS), which, in turn, promote tumor progression and resistance to therapies [170]. The antioxidant glutathione and the transcription factor Nrf2 contribute in balancing ROS levels [170]. As shown in preclinical rat studies, the KD increased the level of glutathione and activated the Nrf2 detoxification pathway [171], [172]. Moreover, in a glioma mouse model, the KD induced antitumor effects and decreased the production of ROS in tumor cells by altering the expression of genes involved in modulating ROS levels and oxidative stress [161], [162].

In contrast, it has been hypothesized that increased ROS production of cancer cells may be compensated by the generation of reducing equivalents through elevated glycolysis and pentose phosphate pathway activity [173]. Thus, limiting the availability of glucose by the KD could selectively induce metabolic oxidative stress in cancer cells. In line with this hypothesis, the combination of a KD and radiation therapy increased the level of oxidative stress and reduced tumor growth in lung and pancreatic cancer-bearing mice [53], [55]. Targeting cancer cells by radiotherapy leads to cellular stress mediated by ROS. However, tumor areas with a lack or low amounts of oxygen are more resistant to radiotherapy than well-oxygenated tumors [174]. Thus, the ROS inducing potential of the KD may explain its additive effects on radiotherapy [53].

Finally, the antitumor effect of the KD has been associated with both increased and reduced ROS levels, indicating that the KD potentially interferes with the tumorigenic ROS balance of cancer cells [175].

## 5 Future potential of the ketogenic diet as an adjuvant cancer therapy

As elucidated in this review, the KD seems to create an unfavorable metabolic environment for cancer cell proliferation and, thus, represents a promising adjuvant for a multifactorial patient-specific therapeutic regime. One clear benefit of the KD is its potential to increase the response to therapeutic drugs, which has been widely demonstrated *in vitro* and *in vivo* [51], [52], [53], [56], [176]. Thus, combining the KD with standard therapy or even novel treatment approaches to enhance the therapeutic response in humans should be a research focus in this field [177]. Limitations of the available evidence (Table 1, Table 2) include heterogeneous study designs and divergent dietary regimes in preclinical as well as clinical studies. For better comparability of the studies, it would be important to have uniform formulations of the KD as well as proper control diets matched in vitamins and minerals. However, since unification of the KD regime in clinical trials might be limited by individual diet preferences of patients, uniform KDs may be feasible only in preclinical studies. Further limitations are the small number of patients plus the lack of proper randomization and control groups within most human studies. Therefore, even though preclinical evidence clearly points toward an overall antitumor effect of the KD, it remains hard to draw generalizable conclusions about the actual effect of the KD on cancer growth and survival in humans [178], [179], [180].

Several clinical trials to elucidate the effect of the KD on cancer patients are ongoing [181] [https://clinicaltrials.gov/], and the results from these trials will be essential to further evaluate the feasibility of the KD in clinical practice. However, future clinical trials with a large number of patients are required to better understand important questions regarding the optimal diet (composition and ratio), calorie consumption as well as the optimal blood glucose and blood ketone concentrations to maximize the antitumor effect [76]. Klement and colleagues initiated a phase I clinical trial investigating whether a part-time KD, for example as a ketogenic breakfast, after radiotherapy delivers similar effects as a complete KD on the body composition of cancer patients [181]. Interim results indicate significant favorable effects of the KD with concurrent radiotherapy on the body composition of colorectal, breast, as well as head and neck cancer patients [88]. Thus, the optimal time point of the KD (before, during and/or after standard therapy) and the stage of disease are further important factors which may influence KD efficacy [79], [84], [177], [181]. Martin-McGill et al. investigated the feasibility and deliverability of a modified KD in glioblastoma patients within the National Health Service in the UK [80]. Interestingly, the study revealed from 172 completed questionnaires that 73% would be willing to try a KD for three months and 66% would be inclined to participate in a clinical study to analyze efficacy and tolerability of the dietary intervention. Moreover, 25% of the respondents stated they would prefer to start the KD before surgery, 22% right after surgery, 15% after surgery during chemotherapy, and 27% after treatment during the monitoring phase [80]. In our opinion, such questionnaire data are highly relevant to guiding the design of clinical trials to maximize their effectiveness.

In conclusion, most preclinical and some clinical studies support the use of the KD as an adjuvant cancer therapy. The mechanisms underlying the KD seem to involve a broad spectrum, ranging from targeting tumor metabolism, gene expression and the tumor microenvironment. To further elucidate the mechanisms behind KD therapy and its use in clinical practice, more molecular as well as well-designed randomized controlled trials are needed. The available scientific literature indicates that, based on controlled preclinical trials, strict KDs could be beneficial in a range of cancers. However, enthusiasm is currently tempered by the lack of clinical trials to warrant routine use of a strict KD as an adjuvant therapy in cancer patients. Nonetheless, it has been shown that a low-carbohydrate diet increased the quality of life in type 2 diabetic patients [182]. Thus, cancer patients may already benefit from moderate low-carbohydrate regimes not leading to a dramatic induction of ketosis without risking adverse effects from the growth of certain types of cancers. In any case, close guidance by clinicians and dieticians is crucial for patients who are changing their diets.
